# The Effect of Microdialysis Catheter Insertion on Glutamate and Serotonin Levels in Masseter Muscle in Patients with Myofascial Temporomandibular Disorders and Healthy Controls

**DOI:** 10.3390/diagnostics9010014

**Published:** 2019-01-22

**Authors:** Ermira Bajramaj, Birgitta Häggman-Henrikson, Andreas Dawson, Björn Gerdle, Bijar Ghafouri

**Affiliations:** 1 Department of Orofacial Pain and Jaw Function, Faculty of Odontology, Malmö University, 205 06 Malmö, Sweden; ermira.bajramaj@gmail.com (E.B.); birgitta.haggman.henrikson@mau.se (B.H.-H.); 2 Department of Odontology, Clinical Oral Physiology, Umeå University, 901 87 Umeå, Sweden; 3 Centre for Oral Rehabilitation, Östergötland County Council, Linköping, 581 85 Norrköping, Sweden; andreas.dawson@regionostergotland.se; 4 Pain and Rehabilitation Centre, and Department of Medical and Health Sciences, Linköping University, 581 85 Linköping, Sweden; bjorn.gerdle@liu.se

**Keywords:** microdialysis, myofascial temporomandibular disorders, serotonin, glutamate, chronic pain

## Abstract

Myofascial temporomandibular disorders (TMD) are the most common cause of chronic pain in the orofacial region. Microdialysis has been used to study metabolic changes in the human masseter muscle. The insertion of the microdialysis probe causes acute tissue trauma that could affect the metabolic milieu and thereby influence the results when comparing healthy subjects to those with TMD. This study aimed to investigate the levels of serotonin and glutamate during the acute tissue trauma period in healthy subjects and in patients with TMD. Microdialysis was carried out in 15 patients with TMD and 15 controls, and samples were collected every 20 min during a period of 140 min. No significant alterations of serotonin or glutamate were observed over the 2 h period for the healthy subjects. For the TMD group, a significant decrease in serotonin was observed over time (*p* < 0.001), followed by a significant increase between 120 and 140 min (*p* < 0.001). For glutamate, a significant reduction was observed at 40 min compared to baseline. The results showed that there was a spontaneous increase of serotonin 2 h after the insertion of the catheter in patients with TMD. In conclusion, the results showed that there are differences in the masseter muscle levels of serotonin and glutamate during acute nociception in patients with myofascial TMD compared to healthy subjects.

## 1. Introduction

Myofascial temporomandibular disorders (TMD) are the most frequent cause of chronic pain in the orofacial region. TMD has a prevalence rate of 10%, and like many other chronic pain conditions, is most common in women [1,2,3]. Patients with TMD typically report muscle pain, soreness, and increased pain during jaw function (e.g., chewing and jaw opening), as well as headaches and psychological distress, such as anxiety, depression, and stress [4,5]. In common with most chronic pain conditions, the pathophysiological mechanism behind TMD is not fully understood. It has been suggested that muscle hyperactivity related to tooth clenching and grinding may cause mechanical overloading, accompanied by disturbed local blood flow resulting in local ischemia and the peripheral release of inflammatory and neuroactive substances, which cause pain [6,7].

Serotonin (5-HT) and glutamate are neurotransmitters thought to be involved in chronic pain [8], and higher concentrations of both have been found in the masseter muscle in patients with myofascial TMD compared to healthy individuals [9,10,11]. Thus, an injection of 5-HT and glutamate can induce pain in healthy individuals and increase pain intensity in patients with myofascial pain. Furthermore, a correlation between pain intensity and an increase of algesic substances has been reported. The peripheral release of 5-HT and glutamate has been investigated by microdialysis in several chronic pain conditions, including TMD [12]. Microdialysis is a well-established technique that allows the sampling of neurotransmitters and algesic substances, as well as the monitoring of time-dependent changes in specific tissue [9,10,13]. It consists of a thin, semi-permeable catheter that mimics a blood vessel, which is inserted in the tissue. The catheter is infused with a perfusate by a mechanical pump and by diffusion down a concentration gradient, which collects the substances that pass through the catheter. The insertion of a probe into the muscle can induce trauma locally in the tissue. The trauma from the needle causes inflammation and other intramuscular alterations, leading to a cascade of intramuscular events, which means that a stabilization phase is needed. To minimize the risk of bias when performing microdialysis experiments, it is important to include a stabilization period, allowing the tissue to recover from possible alteration in the interstitial surroundings [14,15,16,17,18]. Previous microdialysis studies that have applied a stabilization period show that the time spans given vary from 20 to 150 min [19,20,21,22]. The most common stabilization period for a full recovery of the tissue after the puncture trauma (i.e., trauma phase) is 120 min [13,23,24]. However, it may be that the length of the recovery phase after the puncture trauma varies between healthy individuals and individuals with TMD. The aim of this present study is to investigate the necessary stabilization period for microdialysis of serotonin and glutamate in the masseter muscle in healthy subjects and in patients with myofascial TMD. Our hypothesis is that there is a difference between healthy subjects and patients with myofascial TMD regarding the stabilization of 5-HT and glutamate after the trauma phase.

## 2. Materials and Methods

### 2.1. Subjects

Fifteen patients, 11 females and 4 males (mean age 32 years, standard deviation (SD) 10 years), were recruited from consecutive patients referred to the Department of Orofacial Pain and Jaw Function at Malmö University (Malmö, Sweden), and compared to 15 healthy age and gender-matched subjects. Prior to participating in the study, all subjects were examined using the Research Diagnostic Criteria for Temporomandibular Disorders (RDC/TMD) [5]. The inclusion criteria for the patients was a diagnosis of myofascial TMD with at least a 6-month duration (mean duration 59 ± 60 months) using RDC/TMD criteria, with at least moderate pain upon palpation. The inclusion criteria for the healthy subjects was no orofacial pain and the absence of a RDC/TMD diagnosis. The exclusion criteria for both groups were (1) systemic inflammatory connective tissue diseases (e.g., rheumatoid arthritis), (2) chronic widespread muscle pain conditions (e.g., fibromyalgia), (3) neuropathic pain or neurological disorders (e.g., oromandibular dystonia), (4) whiplash-associated disorders, (5) severe skeletal malocclusions, (6) pregnancy or lactation, (7) high blood pressure, (8) anti-coagulants, (9) allergy to antibiotics, (10) prilocaine or lidocaine, (11) the use of analgesics 1 week before the experiment (e.g., paracetamol, NSAIDs, salicylate drugs, and opioids) or (12) other medication that would influence pain perception (e.g., anti-depressants or anti-epileptic drugs), (13) pain of dental origin, (14) extensive restorations (e.g., full bridges, dentures), and (15) ongoing dental treatment. Before enrollment in the study, the patients’ medical history was obtained to assess exclusion criteria, and a questionnaire was completed that explained the duration and intensity of pain assessed on the Numerical Rating Scale.

This study was performed according to the Declaration of Helsinki guidelines and was approved by the Regional Ethics Review Board at Lund University (100119/ Dnr 2009/264). All subjects were given both written and verbal information about the study, and they signed a consent form that was in accordance with the Declaration of Helsinki. All participants received financial compensation after completion of their participation.

### 2.2. Study Design

**Figure 1:**
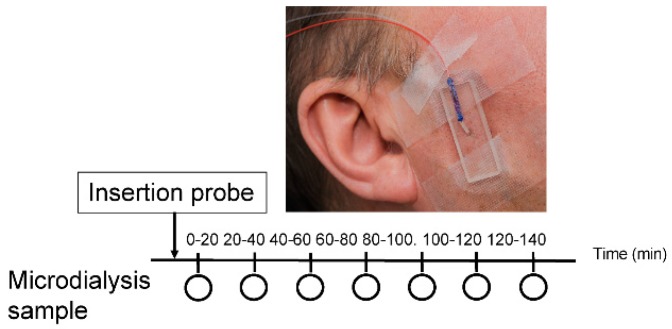
A schematic illustration of the study design. The circle represents every 20 min for sample collection (20, 40, 60, 80, 100, 120 and 140 min). The time point 20 to 100 min indicates the trauma phase and time point 100-120 min indicates the stabilization phase. Intramuscular microdialysis was performed on the right masseter muscle. The microdialysis probe (Ø 0.5 mm; membrane length 10 mm; molecular cut-off: 6 kDa) was inserted into the muscle and perfused at a rate of 5 μL/min with a Ringer-acetate solution.

This experimental study consisted of one session lasting 4 h. Intramuscular microdialysis was conducted in the right masseter muscle to sample interstitial 5-HT and glutamate. A 120 min stabilization period was studied to allow the tissue to recover from possible changes in the interstitial environment due to trauma from probe insertion (Figure 1). The patients were examined in the order in which they signed up for the trial. Subjects sat upright in a dental chair with a head support throughout the experiment and were instructed to relax their masticatory muscles. The masseter muscle was used both due to its clinical importance and its accessibility for the invasive investigations using microdialysis technique. All patients had self-reported pain in the masseter muscle, as well as increased pain on palpation.

### 2.3. Microdialysis

**Figure 2:**
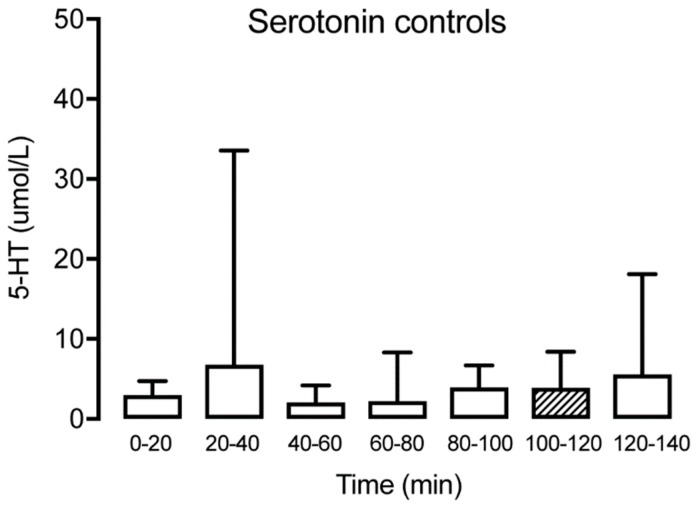
Interstitial serotonin at different time points for the healthy control group (*n* = 15) showing no significant alteration over time. The hatched box indicates time point T100–120, often used as a baseline.

Intramuscular microdialysis was performed to sample masseter 5-HT and glutamate. By palpating the muscle, the most prominent part of the masseter muscle was identified followed by topical anesthetization (EMLA^®^ 20 mg/g; AstraZeneca AB, Södertälje, Sweden) of the skin for 20 min. The EMLA^®^ patch was removed afterwards, and the surface of the skin was cleaned. Thereafter, with a 45° angle to the skin, a standard catheter (Ø 1.3 × 32 mm, BD Venflon Pro; Becton Dickinson Infusion Therapy AB, Helsingborg, Sweden) was directly inserted into the pre-determined region of the masseter muscle at a depth of 20 mm (Figure 2). The needle was later removed, and the catheter was withdrawn, leaving only 10 mm of plastic within the masseter muscle [25]. A sterile microdialysis probe (Ø 0.5 mm; membrane length 10 mm; shaft length 20 mm; molecular cut-off: 6 kDa, MAB11.20.10; Microbiotech/se AB, Årsta, Stockholm, Sweden) was inserted into the masseter muscle via the catheter to an extent of 20 mm measured from the skin surface, making sure that the complete membrane protruded beyond the plastic into the muscle. The probe was then connected to a microdialysis pump (MAB40, Microdialysis Pump Dual Chanel; Microbiotech/se AB). The perfusion rate of the microdialysis probe was at a rate of 5 µL/min, with a Ringer-acetate solution (Baxter Viaflo, Baxter Medical AB, Kista, Sweden) consisting of 3 mM glucose (glucose 50 mg, B. Braun Melsungen AG, Melsungen, Germany) and 0.5 mM Ringer-lactate (Baxter Viaflo, Baxter Medical AB), which prevented the interstitial space from depleting. Three µM [14C]-lactate (specific activity: 7.4 MBq/mL; PerkinElmer Life Sciences, Boston, MA, USA) was added to the Ringer-acetate solution to determine in vivo relative recovery (RR), allowing the determination of the interstitial concentrations of the inflammatory mediators, 5-HT and glutamate [18,26,27]. Intramuscular fluids were sampled every 20 min for a period of 180 min. The samples collected during the stabilization period, between 0 min–140 min, were later analyzed.

### 2.4. Analyses of Algesics Substances and Metabolites

To calculate the concentration of interstitial glutamate and 5-HT, the dialysate and perfusate (5 µL) was pipetted into a counting vial consisting of 3 mL scintillation fluid (High-flash Point, Universal LSC-Cocktail, ULTIMA GOLD™, PerkinElmer, Inc, Boston, MA, USA). The mixture was then vortexed, and β-counting was done in a liquid scintillation counter (Beckman LS 6000TA; Beckman Instruments, Inc., Fullerton, CA, USA). Relative recovery (RR) is a description of the ratio between the concentration of interstitial metabolites and the concentration of the dialysates. For the algesic substances, the levels of interstitial concentration (Ci) were calculated with the following: Ci = (Cd − Cp)/RR + Cp [17,28].

Data collected from the first 140 min was analyzed. Concentrations of glutamate were analyzed with an ISCUS Clinical Microdialysis Analyzer (Dipylon Medical AB, Solna, Sweden). The limit of detection (LOD) for glutamate was 1.0 µmol/L. Half of the LOD was used for samples that were below the limit of detection. The concentration of 5-HT was analyzed with high-pressure liquid chromatography in combination with electrochemical detection [16]. The LOD for 5-HT was 20 fmol/10 µL.

### 2.5. Statistics

The data was analyzed with a GraphPad Prism version 7.0 for Mac, GraphPad Software, La Jolla, CA, USA, www.graphpad.com (Prism). Given that the data was not normally distributed, non-parametric statistics were used to perform the analysis. The Friedman test was used to test the within-group variation in substances over time for each side separately (for glutamate and 5-HT). If significant changes were indicated, a Wilcoxon signed-rank test with Bonferroni correction was used as the post-hoc test to test for significant changes between the various time points. Differences between the TMD and control groups were tested with the Mann–Whitney U test. In Prism, Dunn’s test was performed for multiple comparison of the substances over time. All statistical analyses were performed two-tailed at a significance level of 5% (*p*-value below 0.05 was considered significant).

## 3. Results

### 3.1. Serotonin

**Figure 3:**
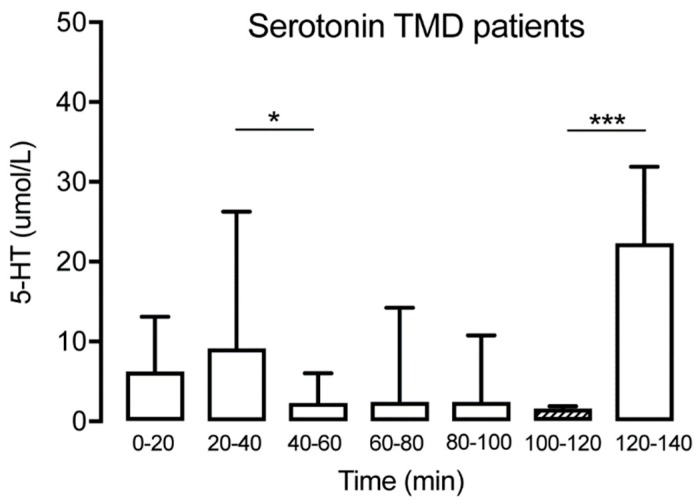
Interstitial serotonin at the different time points for the TMD group (*n* = 15) showing a significant decrease at T40–60 followed by a spontaneous increase at T120–140. The hatched box indicates time point T100–120, often used as a baseline. It was not possible to extract data from 4 of the 15 participants regarding the 5-HT levels at T0-20 due to technical difficulties. * indicates *p* < 0.05 and *** indicates *p* < 0.001.

Serotonin could be detected in microdialysate samples from all time points in patients with TMD and healthy subjects (Table 1). No significant changes of muscle 5-HT were observed over time for the controls (Friedman test: *p* > 0.05, Dunn’s multiple comparisons test *p* > 0.05) (Figure 2). For TMD patients, a significant decrease of muscle 5-HT was observed in time point (T) 40–60 compared to T20–40 (Wilcoxon matched-pairs signed-rank test: *p* < 0.05) but not compared to the trauma phase (T0–20). In general, a significant decrease of muscle 5-HT was observed for the TMD patients over time (Wilcoxon matched-pairs signed-rank test: *p* < 0.001). However, a significant increase between T100–120 and T120–140 for the same group (Wilcoxon matched-pairs signed-rank test: *p* < 0.001) was observed (Figure 3). Compared to the control group, the TMD group had significantly higher levels of 5-HT at T0–20 (*p* = 0.012) and T120–140 (*p* = 0.002), and lower levels at T100–120 (*p* = 0.001).

### 3.2. Glutamate

**Figure 4:**
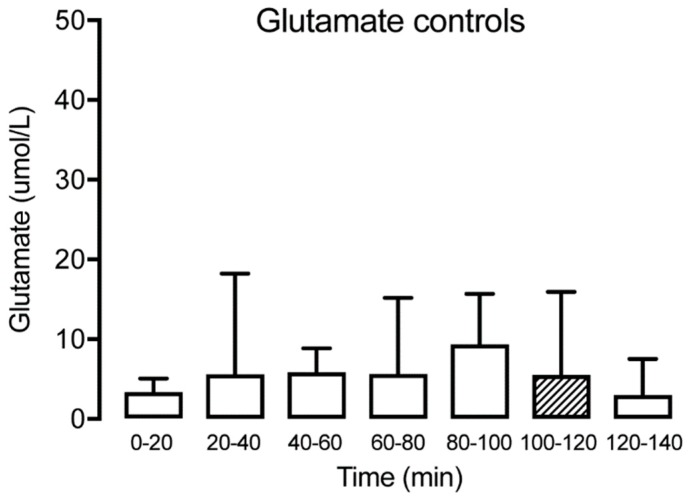
Interstitial glutamate at different time points for the healthy control group (*n* = 15) showing no significant alteration over time. The hatched box indicates time point T100–120, often used as a baseline.

**Figure 5:**
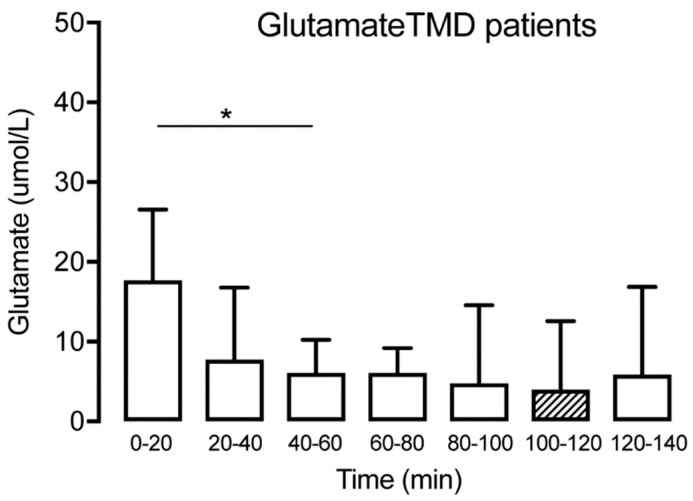
Interstitial glutamate at different time points for the TMD group (*n* = 15) showing a significant decrease until T40–60, followed by a stabilization. The hatched box indicates time point T100–120, often used as a baseline. * indicates *p* < 0.05.

The concentration of glutamate was measured at all time points in patients with TMD and healthy subjects. The levels are expressed as mean (SD) in Table 1. No significant glutamate alterations were observed over time for the controls (Friedman test: *p* > 0.05, Dunn’s multiple comparisons test *p* > 0.05) (Figure 4). For the TMD patients, there was a tendency for a reduction observed at T20–40, but the difference was not significant until T40–60, with a reduction of glutamate compared with the trauma phase T0–20 (Wilcoxon matched-pairs signed-rank test: *p* < 0.05). This was followed by a stabilization of glutamate, with no further significant changes over time (Figure 5). Compared to the control group, the TMD group had significantly higher levels of glutamate at T0–20 (*p* = 0.002).

## 4. Discussion

The main finding of this study is that the myalgic muscle responds differently to acute nociception caused by the insertion of a microdialysis probe, compared to healthy muscle. Surprisingly, the results also indicate that, for TMD patients, a stabilization time of 120 min is not sufficient with regard to 5-HT. Our results suggest that, for healthy individuals, a 20 min stabilization period is sufficient for normalization of both 5-HT and glutamate. However, in patients with myofascial TMD, the normalization of interstitial glutamate after 40 min indicates the need for a longer stabilization period. Furthermore, for 5-HT levels, there was a significant reduction after 100 min, followed by a significant increase after 120 min.

Previous microdialysis studies in TMD patients have reported a significantly higher muscle 5-HT level compared to healthy individuals [14,28], but whether this difference is due to different reaction patterns to the tissue trauma caused by microdialysis probe is not known. Therefore, it was of interest to study the release of 5-HT and glutamate during the commonly proposed trauma period of 0–120 min for metabolites. To the best of our knowledge, there are no studies that have been performed to provide methodological guidance regarding the stabilization time for 5-HT.

Studies have demonstrated that after the insertion of a probe, there is an increase in interstitial 5-HT levels during the trauma phase [11,13,23,24]. Bradykinin is released due to tissue trauma, which can, indirectly, through the B2-receptor, increase the muscle nociceptor’s sensitivity for 5-HT [29]. In response to tissue damage, axon reflexes are evoked that lead to a peripheral release of neuropeptides, such as calcitonin-gene-related peptide and substance P. The neuropeptides facilitate the release of interleukins and cytokines that provoke a release of platelet-activating factors through the degranulation of mast-cells. 5-HT is released due to a platelet-activating factor-induced degranulation of platelets [30].

In order to minimize any risk this effect biasing results, a 2 h stabilization period was suggested. However, the present results show that this is not a sufficient stabilization period, due to the significant increase in interstitial 5-HT levels after 120 min in patients with local myalgia. This spontaneous increase of intramuscular 5-HT might be a side effect of the local anesthesia used before the microdialysis probe implantation. When applying topical anesthesia, the nerve conduction is reversibly blocked near the site of administration, which targets free nerve endings on the dermis leading to a temporary loss of sensation on the applied area. When applying the EMLA-patch for 1 h, it is thought to have an analgesic function for up to 2 h after the removal of the patch. During this study, the topical anesthesia was applied for 20 min, leading to an analgesic effect of approximately 80 min [31]. It is possible that the spontaneous release of intramuscular 5-HT after 2 h occurred due to the decrease of the analgesic effect, leading to a normalization of the depolarization and excitability threshold, which allows nerve endings to generate action potential, thus leading to a sensation of pain and the release of algesic substances. This could be a source of error and a reason for the sudden increase of interstitial 5-HT in the TMD group, but it does not explain why there was not an increase in the control group. However, this could be explained by the earlier suggestion that TMD patients have a significantly higher muscle 5-HT level [28].

The application of the topical anesthesia causes a local vasoconstriction of the superficial arteries, leading to a smaller amount of blood flow and, as a result, a smaller amount of algesic substances are indirectly transported to the affected area [31]. This also means that the intramuscular events following the trauma would take a longer time period to normalize, as a vasoconstriction leads to a slower reduction of waste accumulation. However, in the present study, the insertion of the microdialysis probe was approximately 10 mm deep, and the topical anesthesia does not penetrate the dermis enough to constrict the arteries located at the depth of the inserted probe.

In patients with TMD, it has been suggested that the thrombocyte serotonin transporter-molecules (SERT molecules) have a reduced function. It is possible that both the healthy control group and the TMD patients have approximately the same amount of 5-HT released after trauma, but the SERT molecules are defected in the TMD group, thus causing a disturbance and preventing the reuptake of 5-HT. The results show that the 5-HT levels remained stable throughout the stabilization period for the healthy control group, whereas in TMD patients the levels fluctuated throughout the same time period. The limitation of this study is that we were not able to show when the fluctuation of the 5-HT levels in TMD patients returned to baseline. Further research is needed to investigate the stabilization period for 5-HT after microdialysis catheter insertion. In patients with myofascial TMD, it has been claimed that there is also reduced blood circulation locally [2,32], leading to the inhibition of the reuptake of the algesic substances, which further strengthens the theory that myofascial TMD patients have higher 5-HT levels. The higher levels of 5-HT may indicate that TMD is caused by inflammatory mediators, as it has been reported that 5-HT induces temporomandibular joint nociception by the local release of sympathetic amines and prostaglandins [33].

In summary, it is well known that 5-HT is correlated to pain, but the mechanisms behind it are still unknown, and more research is warranted to further elucidate these mechanisms and the role of muscle 5-HT in the pathophysiology of TMD.

In conclusion, the results show that the insertion of a microdialysis catheter in chronic myalgic muscle causes tissue trauma which influences the results of a microdialysis study. To our knowledge, this is the first study to investigate the differences in the masseter muscle levels of 5-HT and glutamate during the insertion trauma in TMD patients and healthy subjects. This study shows that patients with myofascial TMD and healthy subjects respond differently to an acute nociception caused by microdialysis probe. Analyzing glutamate and 5-HT during tissue trauma may be a useful model to investigate the peripheral molecular mechanisms in myofascial TMD.

## Figures and Tables

**Table 1:** Mean (SD) interstitial serotonin and glutamate at different time points for the healthy control group (*n* = 15) and the myofascial temporomandibular disorders (TMD) group (*n* = 15).

Substances	Subjects	T0–20	T20–40	T40–60	T60–80	T80–100	T100–120	T120–140
Serotonin	Control	3.6 (1.7)	30.0 (59.1)	5.8 (8.2)	8.5 (12.8)	6.0 (7.4)	5.5 (5.0)	8.0 (7.8)
	TMD	8.3 (6.1)	22.7 (36.2)	4.9 (6.1)	12.1 (19.4)	12.1 (23.2)	3.0 (4.5)	27.9 (35.4)
Glutamate	Control	5.8 (6.2)	12.6 (17.5)	7.8 (8.0)	18.0 (40.3)	12.9 (13.7)	12.7 (13.0)	5.2 (4.5)
	TMD	12.0 (3.1)	13.8 (3.6)	4.6 (1.2)	5.6 (1.4)	11.3 (2.9)	5.6 (1.5)	7.2 (1.9)
